# Supplementary material for: Dance displays in gibbons: biological and linguistic perspectives on structured, intentional, and rhythmic body movement
Source: Primates. 2024 Oct 4;66(1):61–73. doi: 10.1007/s10329-024-01154-4 (PMC11735528; doi:10.1007/s10329-024-01154-4)
Supplement: Supplementary file 3 — List of questions included in the survey (PDF 185 KB) [file 10329_2024_1154_MOESM3_ESM.pdf]

1) What is your associated institution / field site? (optional: You may want to also provide your name)

Free-form text response

2) What is your position at your institution?

Options:

Researcher / Curator

Veterinarian

Animal caretaker

other (free-form text response)

3) How long did you monitor / work with gibbons at your institution / site?

Free-form text response

4) How many gibbons did you opportunistically or systematically monitor? Please provide the species and sex of the respective individuals, or give approximations.

Free-form text response

5) In how many individuals did you observe "dances"? Please also note the sex of the respective individuals.

Free-form text response

6) Which was the (approximate) age range of individuals that were observed to "dance"?

Options:

0 - 3 years

3 - 5 years

5 - 7 years

7 - 10 years

10 - 25 years

25 - 35 years

35 + years

Not applicable

7) Have you observed "dances" directed towards ...

Options:

other gibbons

humans

other primate species (neither gibbons nor humans)

self (displacement behavior; no receiver of the dance was apparent)

I have never observed "dances"

8) In which context have you observed "dances" directed towards a gibbon?

Options:

invitation to copulate

socializing / grooming

stressful situation / disturbances

feeding / foraging

other (free-form text response)

9) In which context have you observed "dances" directed towards a human?

Options:

invitation to copulate

socializing / grooming

stressful situation / disturbances

feeding / foraging

other (free-form text response)

10) Did you notice effects of hormonal contraception on the occurence of "dances" in captive female gibbons? If so, please provide details.

Free-form text response

11) Are there any additional observations on gibbon "dance" behavior that you want to share (e.g. frequency of occurence, uniformity of dance patterns)?

Free-form text response

12) Do you have video material of "dances" that you would be willing to share with us for further study (the videos do not need to be recorded at the institution/ field site that you are currently associated with) and / or are you interested in the results of our study? If so, please provide your name and an email address so we can contact you.

Free-form text response
